# Data supporting the growth/no growth interface of *Zygosaccharomyces bailii* in simulated acid sauces

**DOI:** 10.1016/j.dib.2018.10.099

**Published:** 2018-10-27

**Authors:** Aldana L. Zalazar, María F. Gliemmo, Marcelo Soria, Carmen A. Campos

**Affiliations:** a Universidad de Buenos Aires, Facultad de Ciencias Exactas y Naturales, Departamento de Industrias, Buenos Aires, Argentina; b Research Fellow from Consejo Nacional de Investigaciones, Científicas y Técnicas de la República Argentina, Argentina; c Cátedra de Microbiología. Facultad de Agronomía, Universidad de Buenos Aires, INBA-CONICET, Buenos Aires, Argentina; d Member of Consejo Nacional de Investigaciones, Científicas y Técnicas – Instituto de Tecnología de Alimentos y Procesos Químicos (ITAPROQ), Buenos Aires, Argentina

## Abstract

This article contains experimental data, images and methods for the growth/no growth interface of *Zygosaccharomyces bailii* in simulated acid sauces. Mentioned data are related to the research article “Modeling growth/no growth interface of *Zygosaccharomyces bailii* in simulated acid sauces as a function of natamycin, xanthan gum and sodium chloride concentrations” (Zalazar et al., 2018) [1]. The growth was assessed colorimetrically by using 2-(4-iodophenyl)-3-(4-nitrophenyl)-5-phenyl-2H-tetrazolium chloride and 2-methoxy-1,4-naphthoquinone as detection reagents. Furthermore, yeast growth was confirmed by plate count.

**Specifications table**

**Table:** 

Subject area	*Biology, Chemistry.*
More specific subject area	*Food Microbiology*
Type of data	*Table and image*
How data was acquired	*Colorimetric method by using 2-(4-iodophenyl)-3-(4-nitrophenyl)-5-phenyl-2H-tetrazolium chloride (INT) and 2-methoxy-1,4-naphthoquinone (MNQ) as detection reagents. Yeast viability at interfaces was determined by surface plating on Sabouraud agar.*
Data format	*Raw and analyzed*
Experimental factors	*Model systems simulating acid sauces were formulated varying natamycin, NaCl and xanthan gum levels.*
Experimental features	*The visual observation of yeast growth and counts at interfaces were obtained.*
Data source location	*Departamento de Industrias, Facultad de Ciencias Exactas y Naturales, Universidad de Buenos Aires, Ciudad Universitaria, Buenos Aires, Argentina.*
Data accessibility	*Data is provided with this article.*

**Value of the data**

• Photographs provide valuable information about the form of yeast growth in systems containing different levels of stabilizers.
• The data demonstrate that growth/no growth interfaces can be determined by colorimetric methods, being these methods less time consuming than plate count.
• Data obtained from plate counts performed at interfaces allow confirming the validity of the redox technique used.
• The data can be useful for other researchers investigating the growth/no growth interfaces in dispersed systems.

## 1 Data, experimental design, materials and methods

Determination of microbial G/NG interfaces is a useful tool to evaluate microbiological stability and antimicrobial effectiveness. The G/NG boundary of microorganisms can be examined by probabilistic models as a function of the stress factors applied [2]. The combination of stress factors that assure low probability of growth is a key factor to determine product formulation [3]. To evaluate the effect of the stress factors mentioned on yeast growth in acid sauces, different systems were formulated varying natamycin, NaCl and xanthan gum levels, as it is mentioned in Table 1. Model system preparation was described in the research article (Zalazar et al., 2018) [1].

**Table 1:** Concentrations of natamycin, NaCl and xanthan gum in model systems.

**System**^a^	**Natamycin**(mg/L)	**NaCl**(wt%)	**Xanthan gum** (wt%)
A (1–3)	0.0	0.0	0.00
B (1–3)	0.0	0.0	0.50
C (1–3)	0.0	0.0	1.00
D (1–4)	0.0	1.5	0.00
E (1–4)	0.0	1.5	0.50
F (1–4)	0.0	1.5	1.00
G (1–4)	2.0	1.5	0.00
H (1–4)	2.0	1.5	0.50
I (1–4)	2.0	1.5	1.00
D (5–8)	4.0	1.5	0.00
E (5–8)	4.0	1.5	0.50
F (5–8)	4.0	1.5	1.00
G (5–8)	6.0	1.5	0.00
H (5–8)	6.0	1.5	0.50
I (5–8)	6.0	1.5	1.00
J (1–4)	0.0	6.0	0.00
K (1–4)	0.0	6.0	0.50
L (1–4)	0.0	6.0	1.00
M (1–4)	2.0	6.0	0.00
N (1–4)	2.0	6.0	0.50
O (1–4)	2.0	6.0	1.00
J (5–8)	4.0	6.0	0.00
K (5–8)	4.0	6.0	0.50
L (5–8)	4.0	6.0	1.00
M (5–8)	6.0	6.0	0.00
N (5–8)	6.0	6.0	0.50
O (5–8)	6.0	6.0	1.00

a Systems name are related to their position in the microplates shown in Fig. 1, Fig. 2.

### 1.1 Visual observation of yeast growth

Most models have been developed in liquid media, which can successfully mimic the microbial growth environment of liquid foods or dispersed systems with low viscosity, where the microbial growth site is the aqueous phase [4]. However, in solid or semi-solid foods, microorganisms can also grow on the surfaces or within the substrate. In these conditions, microorganisms are immobilized and forced to form colonies [5]. The visual observation of yeast growth in system A (1–3), B (1–3) and C (1–3) was evaluated (Table 1 and Fig. 1). Yeast strain, inocula preparation, viability determination and yeast growth evaluation was described in the research article [1]. To perform this photograph, the wells were inoculated and the growth were observed after 5 days at 25 °C ).

**Fig. 1:**
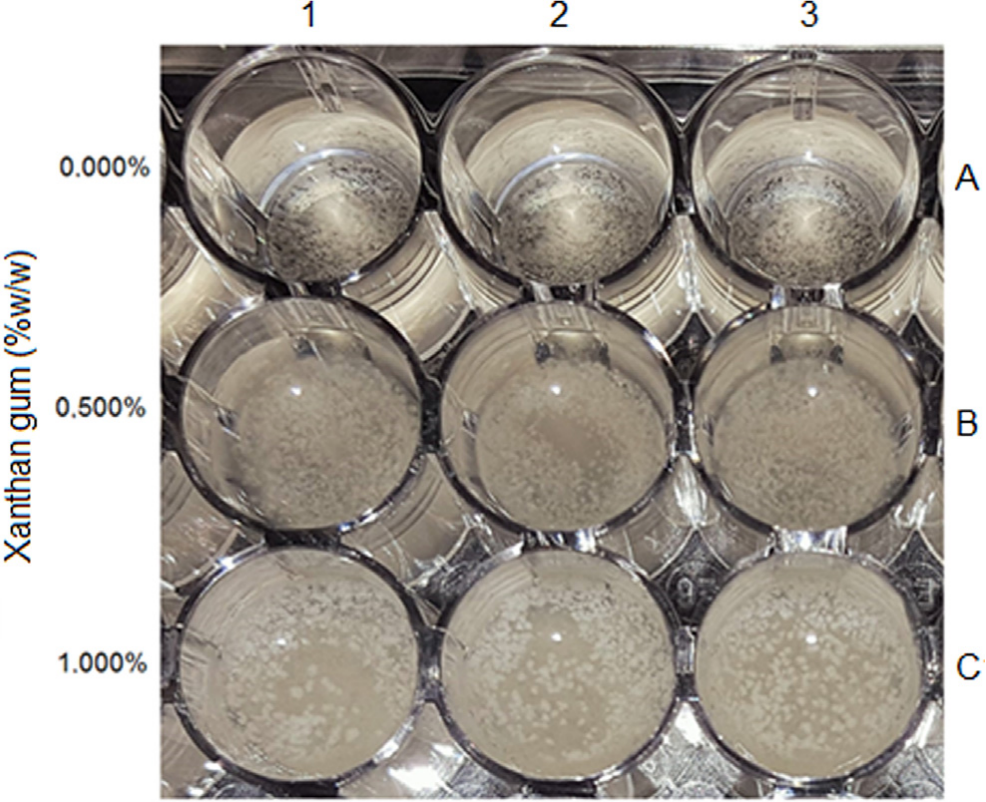
Microplate wells showing the growth of *Z. bailii* in systems containing Sabouraud broth and different concentrations of xanthan gum. Capital letters at each row together with numbers at each column correspond to systems mentioned in Table 1.

**Fig. 2:**
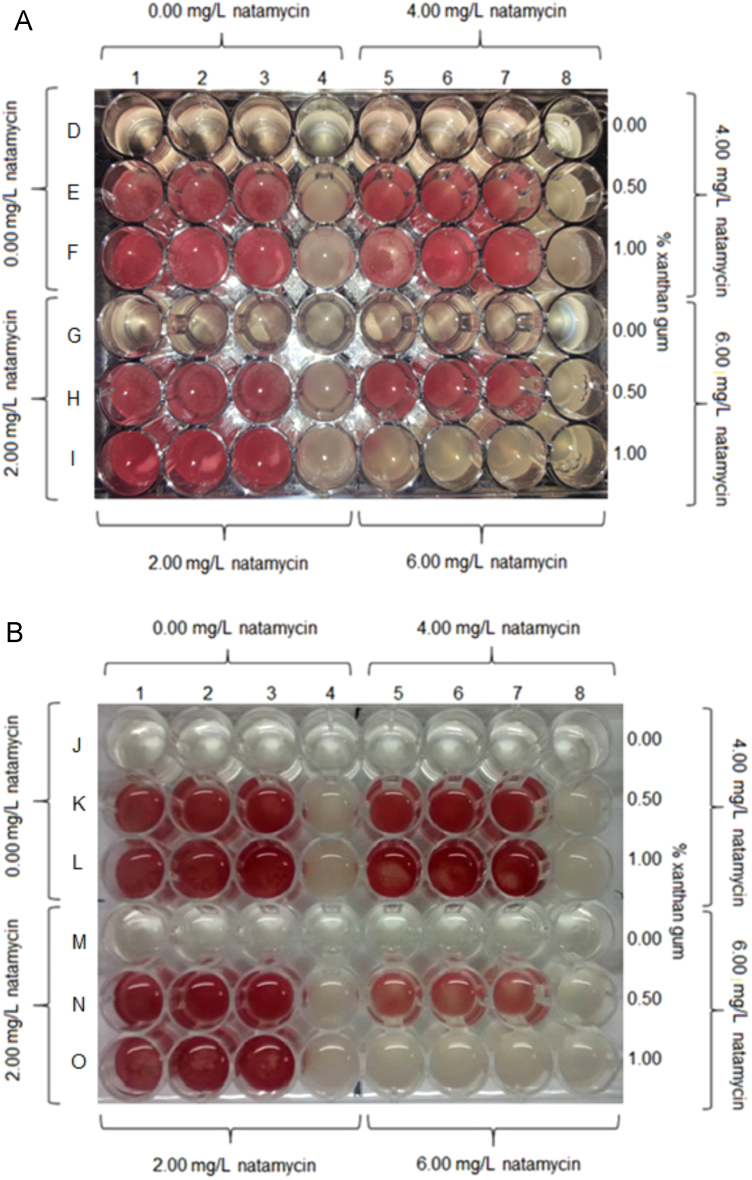
Determination of *Z. bailii* growth/no growth interfaces after 3 h of adding the redox indicator in the presence of 1.50% NaCl (Panel A) and in the presence of 6.00% NaCl (Panel B). Capital letters at each row together with numbers at each column correspond to systems mentioned in Table 1. Columns 4 and 8 without redox indicator (kept for plate count).

### 1.2 Growth/no growth data

The effect of the stress factors on *Z. bailii* on G/NG boundary was determined by a colorimetric method as described in the research article [1]. The visual detection of indicator color change in the wells, as compared with the negative and positive controls, was considered as absence of inhibition. As an example, the results of two microplates are shown in Fig. 2, in which the growth of *Z. bailii* was manifested by the appearance of color in the wells. Furthermore, yeast viability at interfaces was determined by surface plating on SA as described in the research article [1]. Table 2 illustrates the concordance between detection reagent and plate count for the microplates shown.

**Table 2:** *Z bailii* counts at interfaces.

*System*	*Z. bailii population (Log*_*10*_*CFU/ml)* ± *standard deviation*	*System*	*Z. bailii population (Log*_*10*_*CFU/ml)* ± *standard deviation*
D (1–4)	7.60 ± 0.01	J (1–4)	3.63 ± 0.05
E (1–4)	8.28 ± 0.03	K (1–4)	7.43 ± 0.02
F(1–4)	8.24 ± 0.02	L (1–4)	6.83 ± 0.03
G (1–4)	7.88 ± 0.03	M (1–4)	3.18 ± 0.06
H (1–4)	8.55 ± 0.06	N (1–4)	6.78 ± 0.01
I (1–4)	7.47 ± 0.05	O (1–4)	6.76 ± 0.03
D (5–8)	7.50 ± 0.02	J (5–8)	3.32 ± 0.04
E (5–8)	7.27 ± 0.03	K (5–8)	7.31 ± 0.04
F (5–8)	7.42 ± 0.04	L (5–8)	6.44 ± 0.02
G (5–8)	7.60 ± 0.01	M (5–8)	2.60 ± 0.01
H (5–8)	8.48 ± 0.02	N (5–8)	6.39 ± 0.05
I (5–8)	4.00 ± 0.03	O (5–8)	3.42 ± 0.02
